# Inactivation of SREBP-1a Phosphorylation Prevents Fatty Liver Disease in Mice: Identification of Related Signaling Pathways by Gene Expression Profiles in Liver and Proteomes of Peroxisomes

**DOI:** 10.3390/ijms19040980

**Published:** 2018-03-25

**Authors:** Birgit Knebel, Sonja Hartwig, Sylvia Jacob, Ulrike Kettel, Martina Schiller, Waltraud Passlack, Cornelia Koellmer, Stefan Lehr, Dirk Müller-Wieland, Jorg Kotzka

**Affiliations:** 1 Institute of Clinical Biochemistry and Pathobiochemistry, German Diabetes Center at the Heinrich-Heine-University Duesseldorf, Leibniz Center for Diabetes Research, Aufm Hennekamp 65, 40225 Duesseldorf, Germany; bknebel@ddz.uni-duesseldorf.de (B.K.); sonja.hartwig@ddz.uni-duesseldorf.de (S.H.); Sylvia.Jacob@ddz.uni-duesseldorf.de (S.J.); Ulrike.Kettel@ddz.uni-duesseldorf.de (U.K.); Martina.Schiller@ddz.uni-duesseldorf.de (M.S.); Waltraud.Passlack@ddz.uni-duesseldorf.de (W.P.); Cornelia.Koellmer@ddz.uni-duesseldorf.de (C.K.); Stefan.Lehr@ddz.uni-duesseldorf.de (S.L.); 2 German Center of Diabetes Research Partner, 40225 Duesseldorf, Germany; 3 Clinical Research Centre, Department of Internal Medicine I, University Hospital Aachen, 52074 Aachen, Germany; dirmueller@ukaachen.de

**Keywords:** phosphorylation of SREBP-1a, hepatic gene expression, peroxisome proteome, phosphorylation in lipid metabolism, liver peroxisomes, metabolic syndrome, fatty liver, olfactory receptors

## Abstract

The key lipid metabolism transcription factor sterol regulatory element-binding protein (SREBP)-1a integrates gene regulatory effects of hormones, cytokines, nutrition and metabolites as lipids, glucose, or cholesterol via phosphorylation by different mitogen activated protein kinase (MAPK) cascades. We have previously reported the impact of SREBP-1a phosphorylation on the phenotype in transgenic mouse models with liver-specific overexpression of the N-terminal transcriptional active domain of SREBP-1a (alb-SREBP-1a) or a MAPK phosphorylation site-deficient variant (alb-SREBP-1a∆P; (S63A, S117A, T426V)), respectively. In this report, we investigated the molecular basis of the systemic observations by holistic analyses of gene expression in liver and of proteome patterns in lipid-degrading organelles involved in the pathogenesis of metabolic syndrome, i.e., peroxisomes, using 2D-DIGE and mass spectrometry. The differences in hepatic gene expression and peroxisomal protein patterns were surprisingly small between the control and alb-SREBP-1a mice, although the latter develop a severe phenotype with visceral obesity and fatty liver. In contrast, phosphorylation site-deficient alb-SREBP-1a∆P mice, which are protected from fatty liver disease, showed marked differences in hepatic gene expression and peroxisomal proteome patterns. Further knowledge-based analyses revealed that disruption of SREBP-1a phosphorylation resulted in massive alteration of cellular processes, including signs for loss of targeting lipid pathways.

## 1. Introduction

Lipotoxicity due to ectopic lipid accumulation is the most critical condition in obesity [1]. It resembles the systemic overflow with increased fluxes of plasma free fatty acids (FFA) and triglycerides (TG) leading to ectopic lipid accumulation, alteration of tissue glucose metabolism, and blood glucose clearance, resulting in obesity-associated insulin resistance in non-adipose tissues like the liver.

Besides dietary factors, elevated levels of FFA and TG can also be due to increased hepatic de novo lipid synthesis (DNL) [2,3]. The key transcription factors of lipid metabolism governing DNL are the sterol regulatory element-binding proteins (SREBPs), which were initially identified as cholesterol sensors for LDL receptor gene expression [4,5,6]. There are two different genes coding for SREBP-1 and SREBP-2 isoforms, whereas SREBF-1 is further transcribed into two splice variants, SREBP-1a and SREBP-1c [7]. While SREBP-2 mainly regulates cholesterol synthesis, the isoform SREBP-1c controls the synthesis of fatty acids. In contrast, the isoform SREBP-1a is involved in both pathways [8,9]. Transcriptional inactive SREBP precursors are embedded in the membrane of the endoplasmic reticulum, as part of the intracellular endomembrane system. A complex proteolytic machinery releases the mature N-terminal transcriptional active SREBP domains, which translocate into the nucleus [4,5,6]. Furthermore, trans-activity of the mature SREBPs is post-transcriptionally regulated by interaction of phosphorylation by MAPK cascades, protein stability, or degradation by e.g., GSK3 phosphorylation or SUMOylation [10,11,12,13,14,15]. The relevant amino acids of this posttranslational regulatory network are either identical or in close proximity [15]. Next to these posttranscriptional modifications, SREBPs, like other transcription factors of the basic helix-loop-helix family, bind as homo- or heterodimers to the corresponding promoter elements to initiate transcription.

SREBP-1a, target of stimuli-specific differential phosphorylation by ERK-, JNK-, or p38 MAP kinases [10,11,12], mediates gene regulatory effects of hormones, cytokines, nutrition and metabolites as lipids, glucose as well as cholesterol. We have formerly reported the systemic impact of phosphorylation on this central regulator of lipid metabolism in vivo in transgenic mouse models expressing the N-terminal transcriptional active domain of SREBP-1a or a phosphorylation-deficient variant with all MAPK kinase (ERK-1/-2, JNK and p38 stress kinase (S63A, S117A, T426V)) phosphorylation sites mutated tissue specifically the in liver [12]. Overexpression of SREBP-1a in liver resulted in massively increased amount of visceral adipose tissue featuring a fatty liver with hepatic lipid accumulation in mice. This phenotype was abolished in the phosphorylation-deficient model, although, despite not getting obese, the SREBP-1a phosphorylation site-deficient mice animals develop a mild fatty liver [12].

Hepatic lipid accumulation can be prevented by intensified lipid degradation in mitochondria and, to a certain specialized extent, in peroxisomes. Peroxisomal lipid metabolism differs from mitochondrial lipid metabolism in regard to substrate specificity, unregulated substrate concentration-related lipid influx into the organelle and energy net balance [16]. This might act as an emergency reserve metabolism in states of hepatic lipid overflow. In this context, we have shown that, in a model of metabolic syndrome with fatty liver and obesity, the hepatic peroxisomes are the first organelle to alter functionality, whereas mitochondrial functionality was affected when peroxisomes already started to perish in a more diseased model of non-alcoholic fatty liver disease [17,18].

In the present study, we investigated the molecular basis of the systemic observation in regard to hepatic gene expression alterations and lipid degrading organelle function. To determine the physiological impact of this observation we analyzed the hepatic gene expression and the peroxisomal proteome pattern by DIGE and mass spectrometry. We show that (i) hepatic gene expression and peroxisomal proteome are altered most remarkable in the phosphorylation-deficient model of SREBP-1a; (ii) SREBP-1a centered signaling, hormonal regulation, and overall transcriptional regulation were altered due to phosphorylation; and (iii) peroxisomal activity is depending on phosphorylation state of SREBP-1a.

## 2. Results

### 2.1. Influence of SREBP-1a Phosphorylation on the Phenotype

We have previously reported [12], that liver specific overexpression of SREBP-1a resulted in altered body composition with massive adipositas and the development of an enlarged fatty liver under normocaloric diet. Mice were not hyperphagic and consumed food comparable to controls, but the overall the weight gain per food consumed was increased. Adipose tissue mass was increased due to hyperplasia and there were no signs for adipocyte hypertrophy or increased inflammation. In contrast, the phosphorylation-deficient alb-SREBP-1aΔP mice were approximately 10% smaller and voluntarily consumed approximately 20% less food than C57Bl6 and had a weight gain of only 30%.

**Figure 1:**
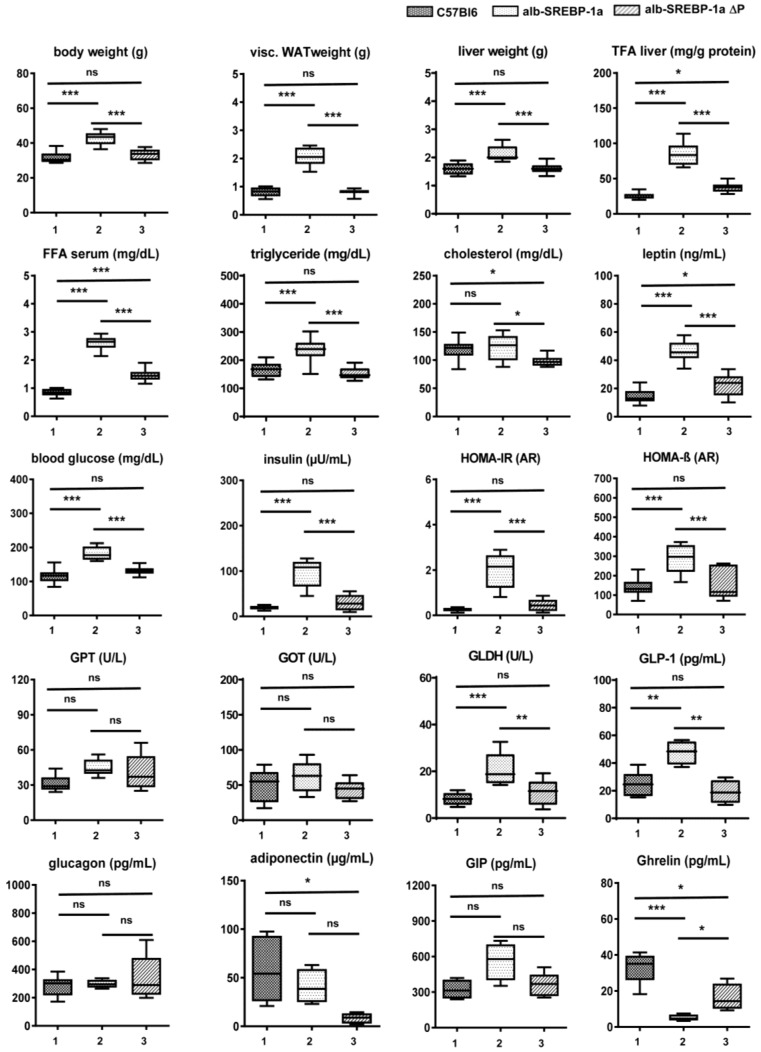
Metabolic characterization of (1) C57Bl6, (2) alb-SREBP-1a and (3) alb-SREBP-1a∆P mice used in the study. Clinical parameters were determined in male mice at 24 weeks of age. Data are expressed as mean ± SD (*n* = 8 of each phenotype). * *p* < 0.05, ** *p* < 0.01, *** *p* < 0.001 by Student’s *t* test. Diagram title indicates parameter displayed on *Y*-axis. Abbreviations are: FFA, free fatty acids; GLDH, glutamate dehydrogenase; GIP, glucagon like peptide; GPT, glutamat-pyruvat-transaminase; GOT glutamat-oxalacetat-transaminase; HOMA-IR, homeostatic model assessment of insulin resistance; HOMA-%β, homeostatic model assessment of β-cell function (%); TFA, total fatty acids; Visc. WAT, visceral white adipose tissue.

The clinical parameters of the mouse models introduced in the present study at 24 weeks of age were summarized in Figure 1. As previously reported [12], the alb-SREBP-1a mice developed visceral obesity, fatty liver, and dyslipidemia characterized by increased serum lipids, i.e., triglycerides (TG), but not cholesterol, indicating insulin resistance. In accordance to that, plasma glucose and insulin levels were increased. Interestingly to note, levels of glucagon like peptide (GLP-1) were also elevated. In contrast, the alb-SREBP-1aΔP mice were similar to controls but had slightly elevated hepatic total fatty acids (TFA), increased serum free fatty acids (FFA) and a hypocholesterinemia. Liver enzymes glutamat-oxalacetat-transaminase (GOT) and glutamat-pyruvat-transaminase (GPT) were numerically increased, although not statistically significant. Elevated glutamate dehydrogenase (GLDH), indicating beginning liver injury, was elevated in alb-SREBP-1a but not in alb-SREBP-1aΔP (Figure 1). Differences in leptin or adiponectin corresponded to the degree of obesity in the models or indicated functional regulation of food intake (ghrelin) (Figure 1).

Insulin sensitivity, indicated as HOMA-IR, correlated to blood glucose (BG) as well as insulin in C57Bl6 and alb-SREBP-1a mice (BG (*r* = 0.778, *p*-value = 0.023), (0.756, 0.03); insulin (0.886, 3.4 × 10^−3^); (0.947, 3.5 × 10^−3^) (Table S1)). In alb-SREBP-1aΔP mice, HOMA IR was not related to BG, but to insulin (0.995, 3.0 × 10^−7^).

The β-cell function (HOMA-β%) negatively correlated to BG (−0.822, 0.012) in C57Bl6. In both transgenic models, it correlated to insulin (alb-SREBP-1a: 0.791, 0.02; alb-SREBP-1aΔP: 0.975, 3.8 × 10^−5^) and further to insulin sensitivity in alb-SREBP-1aΔP (0.949, 3.0 × 10^−4^).

Of lipid parameters, FFA correlated to BG levels (0.880, 4.0 × 10^−4^) and insulin sensitivity (0.807, 0.016) in the controls, but not in both transgenic models. Cholesterol negatively correlated to GOT in controls (−0.733, 0.038), and negatively to hepatic fat content (TFA) (−0.818, 0.013) and TG (0.896, 2.6 × 10^−3^) in alb-SREBP-1a. Despite hypocholesterinemia in alb-SREBP-1aΔP, no correlation was observed here. Of liver parameters, and GLDH correlated to GOT (−0.714, 0.046) in C57Bl6 and to TFA (0.809, 0.015) in alb-SREBP-1a, whereas in the phosphorylation-deficient alb-SREBP-1aΔP GOT correlated to TG (0.749, 0.033) and TFA (0.879, 0.004).

**Figure 2:**
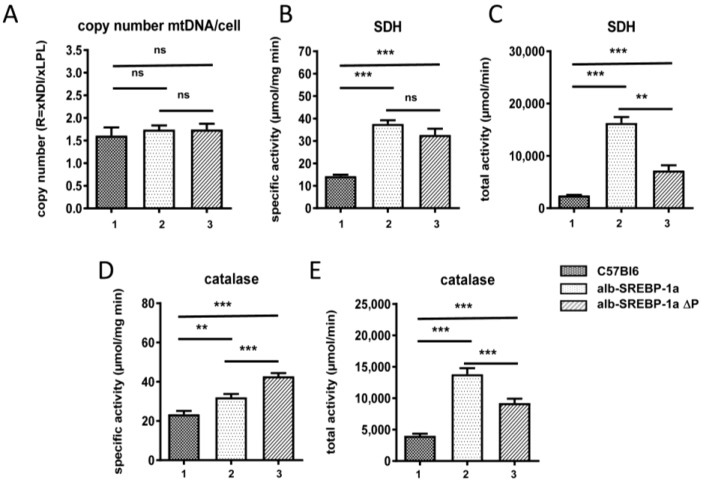
Mitochondria and Peroxisomes in (1) C57Bl6, (2) alb-SREBP-1a and (3) alb-SREBP-1a∆P mice. (**A**) The mitochondrial DNA content was determined in comparison to genomic DNA in C57Bl6, alb-SREBP-1a and alb-SREBP-1a∆P mice (*n* = 15). Mitochondrial SDH activities (specific (**B**), total (**C**)) and specific (**D**) and total (**E**) peroxisomal catalase activity were determined in liver homogenates of C57Bl6, alb-SREBP-1a and alb-SREBP-1a∆P mice (*n* = 15). Data are expressed as mean ± SD (*n* = 8 of each phenotype). * *p* < 0.05, ** *p* < 0.01 *** *p* < 0.001 by Student’s *t* test. Abbreviations are: mtDNA, mitochondrial DNA; SDH, succinate dehydrogenase.

In regard to lipid degrading organelles, the total number of mitochondria determined by mitochondrial DNA copy number indicated no difference in all mouse models (Figure 2). The specific SDH activity was increased in alb-SREBP-1a and in SREBP-1aΔP to the same extend, indicating no influence whether SREBP-1a can be phosphorylated at MAPK sites, or not (Figure 2). The total SDH activity depended on the liver weight was highest in alb-SREBP-1a mice. In C57Bl6 specific SDH correlated positively to liver-weight (0.899, 2.38 × 10^−3^), whereas total SDH correlated to GPT (0.949, 3.28 × 10^−4^) and TFA (0.737, 0.037). In alb-SREBP-1a, a negative correlation of total SDH to liver weight (0.907, 1.88 × 10^−3^) and a positive correlation to adipose tissue occurred (−0.893, 2.79 × 10^−3^). Of note, liver weight and adipose tissue showed negative correlation in this genotype (−0.842, 8.74 × 10^−3^). In alb-SREBP-1a∆P specific SDH correlated positive to liver weight (0.956, 2.07 × 10^−4^) as in the controls and total SDH positive to catalase activity (0.939, 5.52 × 10^−4^).

In contrast to mitochondrial function, the ability to phosphorylate SREBP-1a at MAPK sites had an impact on peroxisomal function. The specific activity of peroxisome marker enzyme catalase was increased in alb-SREBP-1a compared to C57Bl6 mice. This was further pronounced in mice with the phosphorylation-deficient SREBP-1aΔP, focusing on peroxisomes as main physiological target as mediators of the phosphorylation effect of SREBP-1a. Again, total catalase activity depending on the increased liver weight was highest in alb-SREBP-1a.

Of clinical parameters, in C57Bl6 a negative correlation of specific and total catalase (−0.792, 0.019; −0.709, 0.019) to cholesterol was determined (Table S1), which was lost in alb-SREBP-1a and alb-SREBP-1a∆P. Catalase activity in alb-SREBP-1a correlated positively to FFA content (0.745, 0.034), and in alb-SREBP-1a∆P a negative correlation with the amount of visceral adipose tissue (−0.862, 0.006) was determined.

### 2.2. Role of Functional MAPK-Related Phosphorylation Sites in SREBP-1a for Hepatic Gene Expression

The numbers of differential regulated transcripts in the comparisons C57Bl6 vs. alb-SREBP-1a, C57Bl6 vs. alb-SREBP-1a∆P, and alb-SREBP-1a vs. alb-SREBP-1a∆P with the top 15 significant regulated genes were summarized in Table 1 (complete expression analyses in Table S2).

**Figure 3:**
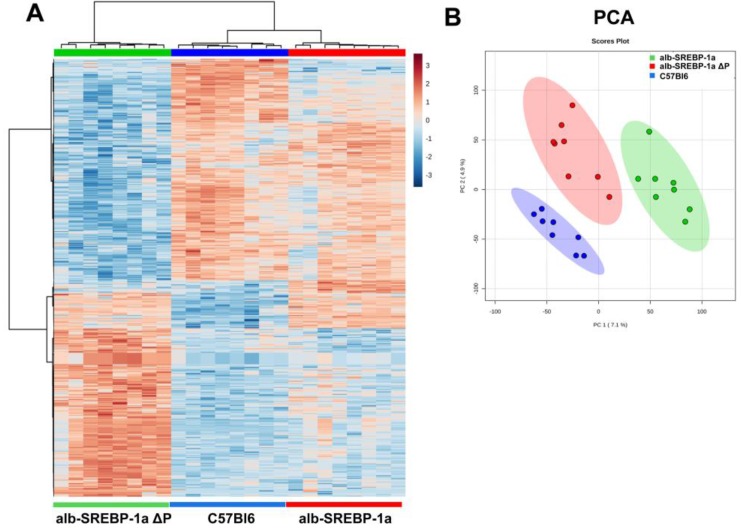
Hepatic gene expression in C57Bl6, alb-SREBP-1a and alb-SREBP-1a∆P mice. (**A**) Heat map of differential expressed genes (top 2500 genes, ANOVA, complete analyses in Table S2). (**B**) Principal component analyses (PCA) of all expressed genes.

Although of similar phenotype, the comparison of C57Bl6 and alb-SREBP-1a∆P identified the most differences on hepatic gene expression. Compared to C57Bl6, in alb-SREBP-1a as well as alb-SREBP-1a∆P, differential expression of NR1D1 was observed as one of the top hits (Table 1). The ability to phosphorylate SREBP-1a further interfered with the hepatic gene expression in direct comparison to functional SREBP-1a. The highest significant regulation in alb-SREBP-1a include e.g., Acadl (acyl-Coenzyme A dehydrogenase, long-chain) whereas predominant regulated genes in alb-SREBP-1a∆P were of unknown function. Overall, the hepatic gene expression patterns influenced by overexpression of SREBP-1a and SREBP-1a deficient in phosphorylation at MAPK sites indicated significant differences to clearly discriminate the genotypes (Figure 3).

**Figure 4:**
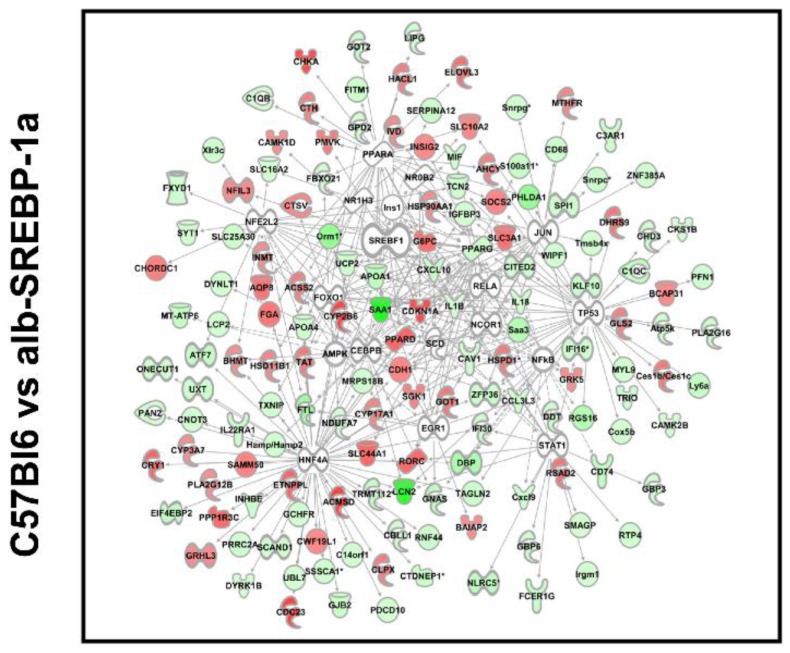

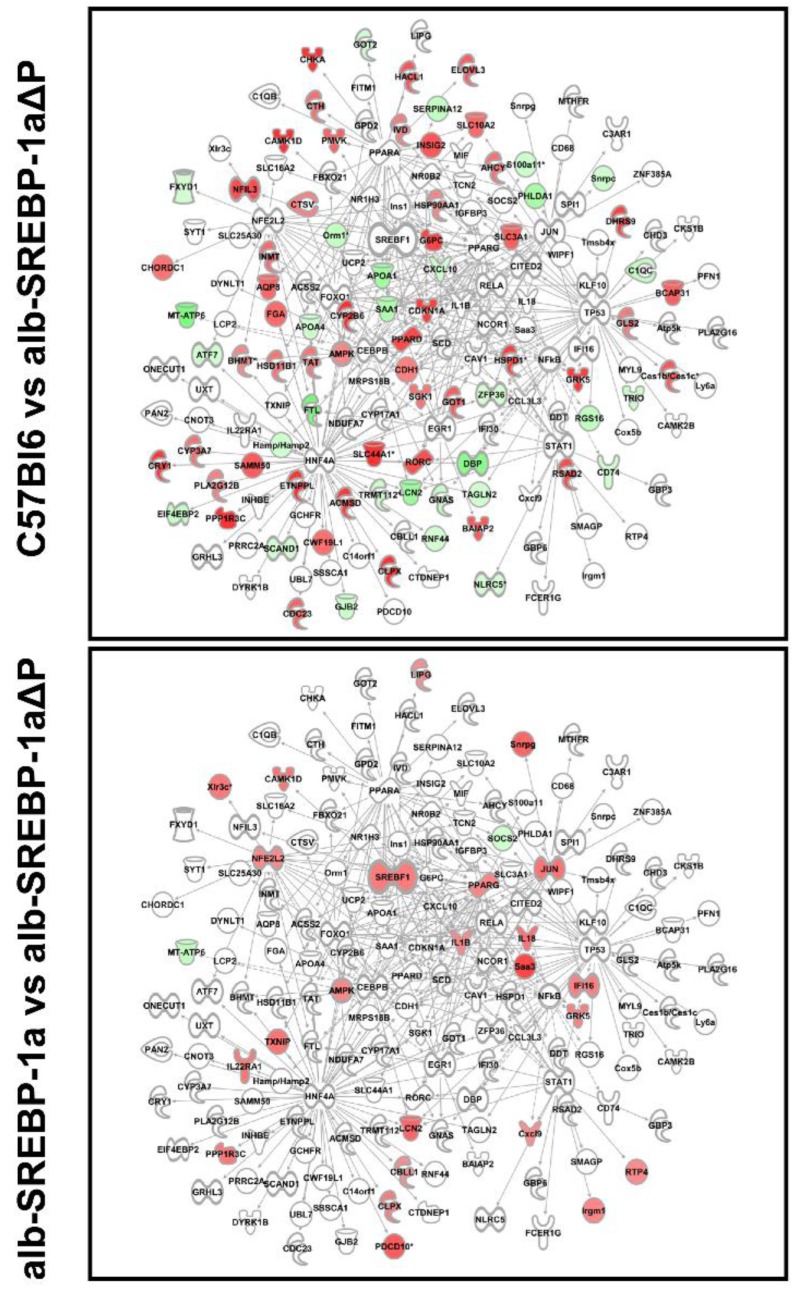
Differential regulation of SREBP-1-centered genes in the comparisons C57Bl6 vs. alb-SREBP-1a, C57Bl6 vs. alb-SREBP-1a∆P, and alb-SREBP-1a vs. alb-SREBP-1a∆P. Genes with differential gene expression (1.5-fold, *p*-value < 0.05) were used for IPA Core analyses. Genes in the SREBP-1 network were analyzed for differential expression in the comparisons C57Bl6 vs. alb-SREBP-1a, C57Bl6 vs. alb-SREBP-1a∆P, and alb-SREBP-1a vs. alb-SREBP-1a∆P. Color code indicates: red: increase in condition 1, green: decrease in condition 1 based on measured expression differences.

We next focused on molecules centered on SREBP-1 in bioinformatics analyses. The comparisons indicated specific expression differences, and phosphorylation pronounced this (Figure 4). SREBP-1-centered genes with differential abundance in C57Bl6 vs. alb-SREBP-1a (further referred as: SREBP-1a subnet) included e.g., *Elovl3*, *Slc3A1*, *Insig2*, *Ppard*, *G6pc* to be upregulated in C57Bl6 and *Saa1*, *ApoA4*, *ApoA1*, *Irgm1*, *Atf7*, *Pparg* to be upregulated in alb-SREBP-1a.

SREBP-1-centered genes with differential abundance in C57Bl6 vs. alb-SREBP-1a∆P (further referred as: SREBP-1a∆P subnet) indicated the transcription of *Ppard*, *Slc16 2A*, *G6pc*, *AMPK* components to be upregulated in C57Bl6 and *Lcn1*, *Saa1*, *ApoA1*, *ApoA4*, *Atf7*, *Got* upregulated in alb-SREBP-1a∆P. SREBP-1-centered genes with differential abundance in the direct comparison of alb-SREBP-1a vs. alb-SREBP-1a∆P (also referred to as the phosphorylation subnet) showed *Jun*, endogenous *Srebp-1*, *Pparg*, *Slc16 2A*, *AMPK* components to be upregulated in alb-SREBP-1a, and *Socs2*, *Mt-Atp5* to be higher expressed in alb-SREBP-1a∆P.

As gene expression data confirmed alterations in regard to the centered molecule SREBP-1 and its ability to be phosphorylated, further functional annotations were performed to determine SREBP-1 phosphorylation dependent actions on overall hepatic gene expression.

### 2.3. Overall Hepatic Gene Expression Differences in C57Bl6 vs. alb-SREBP-1a 

In this comparison, the differential abundant transcripts end up to e.g., FXR/RXR, PPAR, or sirtuin signaling pathways (examples given in Table 2; complete analyses in Table S3). Upstream regulators with the highest impact were RORA, RORC, PPARA, PPARD, GPD1, SLC25A13, and HNF4A. On functional level, there was overlap to hepatic steatosis associated pathways, liver cholestasis, hyperplasia/hyperproliferation or proliferation, renal damage, or nonalcoholic fatty liver disease. Overall, there was an increased level of SREBP-1a expression and all actions were more or less expected from the previous knowledge on SREBP-1.

### 2.4. Overall Hepatic Gene Expression Differences in C57Bl6 vs. alb-SREBP-1a∆P 

Despite the high number of regulated transcripts, the overlap to canonical pathways or informative networks was low in this comparison (examples given in Table 2; complete analyses in Table S4). They included amino acid metabolism like methionine, isoleucine, or valine degradation, cysteine biosynthesis, general degradation pathways, like autophagy, protein ubiquitination, or sirtuin signaling. Metabolic pathways also showed low enrichment for ceramide biosynthesis, and fatty acid β-oxidation.

The differential regulated transcripts identified upstream regulators like transcription factors HNF4A, RORC, RORA, ONECUT1, MYC, NR1D1, or peroxisome-related ACOX1 (peroxisomal acyl-CoA oxidase), PPARA, and RXRA. The most probable pathological alteration occurred in interacting networks centered to ACOX1, GRB14, and PPARGC1A. Metabolic centered networks indicated differential expression in amino acids metabolism, oxidation of lipid or fatty acids, and fatty acid metabolism as the main pathological target. Putative functions focused on liver dysfunction e.g., bleeding of liver, compensated cirrhosis, degeneration, or cholestasis.

In the differential regulated transcripts of this comparison, a vast number of transcripts (*n* = 514) coded for olfactory receptors (OLR), the largest gene family in the genome, or the related vomeronasal gene family (*vmn*) (*n* = 151). As a consequence, olfactory response or olfaction gives the highest scores in disease-relevant view on the expression data, followed by signal transduction and cell communication. Furthermore, RNA post-transcriptional modification, synthesis of ribonucleoside monophosphate pointed towards disturbances in general transcriptional control.

### 2.5. Overall Hepatic Gene Expression Differences in alb-SREBP-1a vs. alb-SREBP-1a∆P 

Like in the previous comparison, the overlap of differential regulated transcripts to distinct pathways was limited (examples given in Table 2; complete analyses in Table S5). Enrichment of RNA polymerase II complex assembly, protein ubiquitination pathways, sirtuin signaling, or SUMOylation components might indicate alterations in cellular growth and turnover. More informative was the search for upstream regulatory molecules like transcription factors HNF4A, RORC, RORA, or peroxisomal-associated PPARA, or ACOX1. Key signaling networks centered around NCAM1, ACOX1, SIRT4, NR2F1, FOXO4, FABP1, ABCB1, or PPARA. This could be summarized to increased clearance of bilirubin, proliferation of hepatic stellate cells, or hepatomegaly as pathophysiological complications.

Again, in this comparison OLR and vmn gene families were highly represented (*n* = 416; *n* = 120). According to the OLR genes again olfactory response, olfaction, signal transduction and cell communication, had the highest overlap. Nevertheless, RNA post-transcriptional processing, RNA transport or differentiation processes were also abundant.

**Figure 5:**
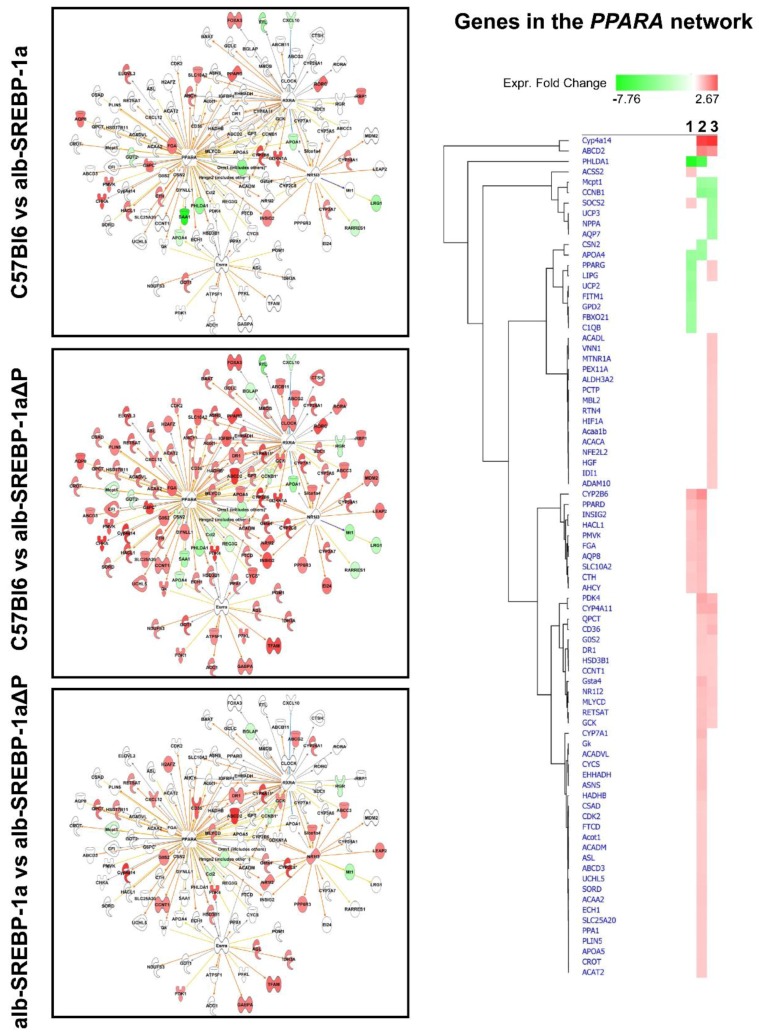

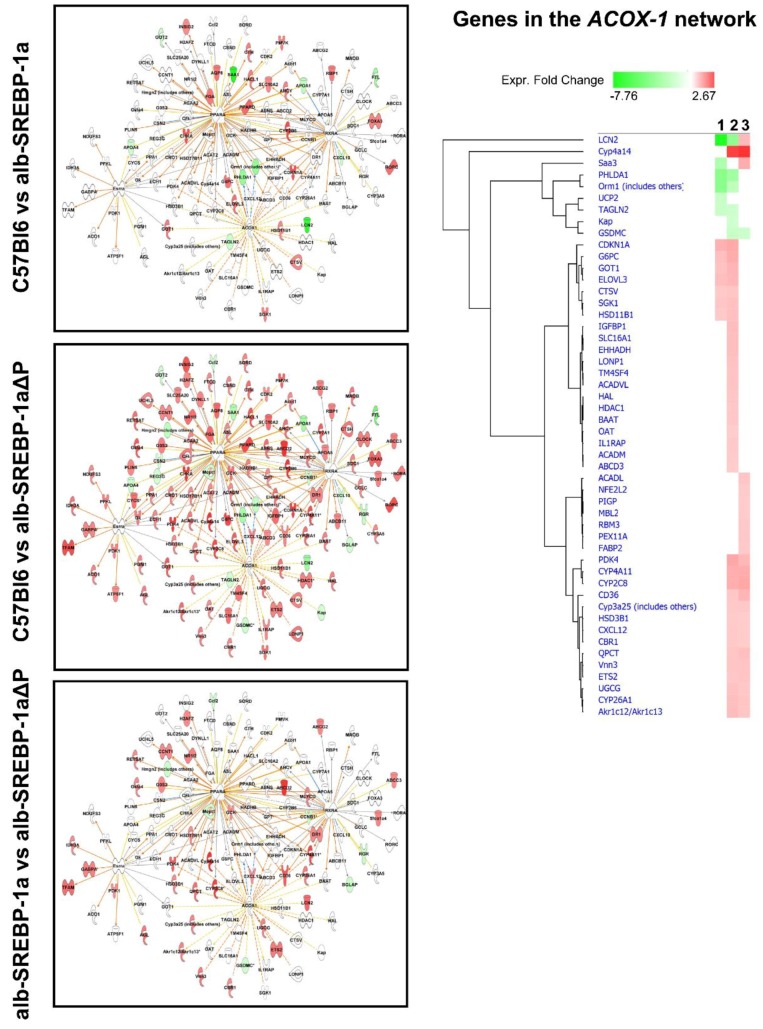
Overlap of the differential hepatic gene expression of the comparisons C57Bl6 vs. alb-SREBP-1a, C57Bl6 vs. alb-SREBP-1a∆P, and alb-SREBP-1a vs. alb-SREBP-1a∆P to interaction networks centered to PPARA or ACOX-1. Genes with differential gene expression (1.5-fold, *p*-value < 0.05) were used for IPA core analyses. Color code indicates knowledge-based interpretation (amber: predicted activation, blue: predicted inhibition, yellow: inconsistence with state of downstream molecule, black: effect not predicted) and different abundance (red: increase in condition 1, green: decrease in condition 1) based on measured expression. Different abundance of network genes is indicated in detail in the hierarchical cluster with 1: C57Bl6 vs. alb-SREBP-1a, 2: C57Bl6 vs. alb-SREBP-1a∆P, and 3: alb-SREBP-1a vs. alb-SREBP-1a∆P.

Taken together, the general annotation of the differential gene regulation came up with the same pathways. Nevertheless, there were specific differences in the molecules regulated in the three comparisons. Although there was overlap in the identified keywords and functions, the detailed look on the processes indicated the specifics as indicated in the example PPARA or ACOX-1 (Figure 5).

### 2.6. Systemic Influence of SREBP-1a Phosphorylation on Hepatic Gene Expression

**Figure 6:**
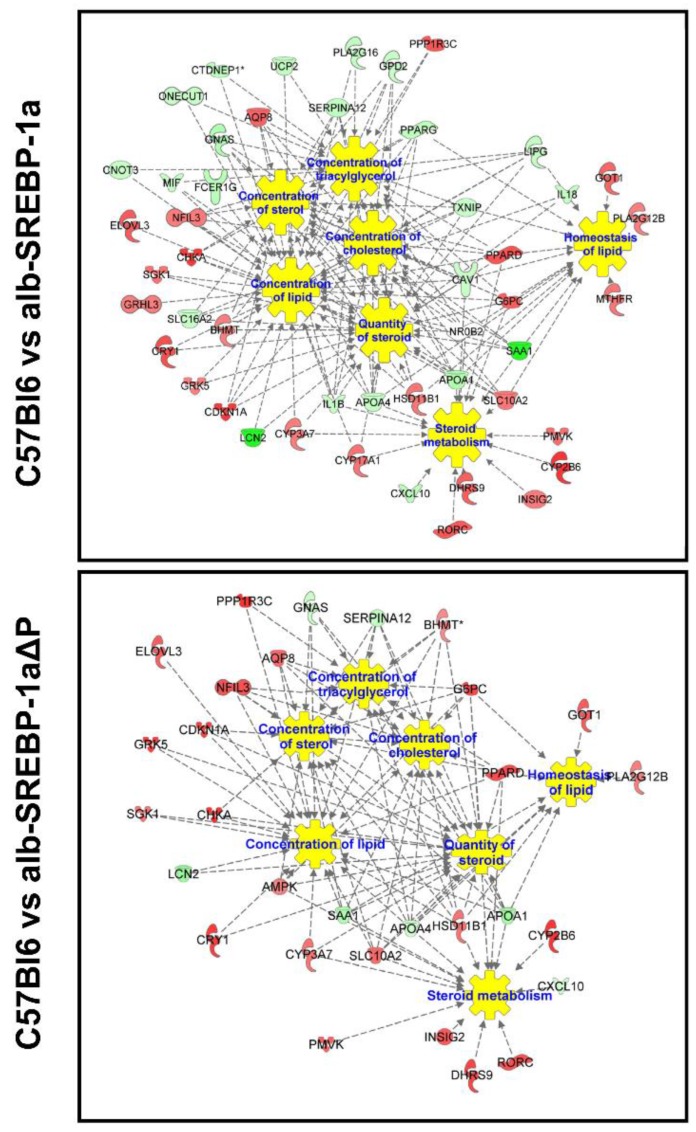

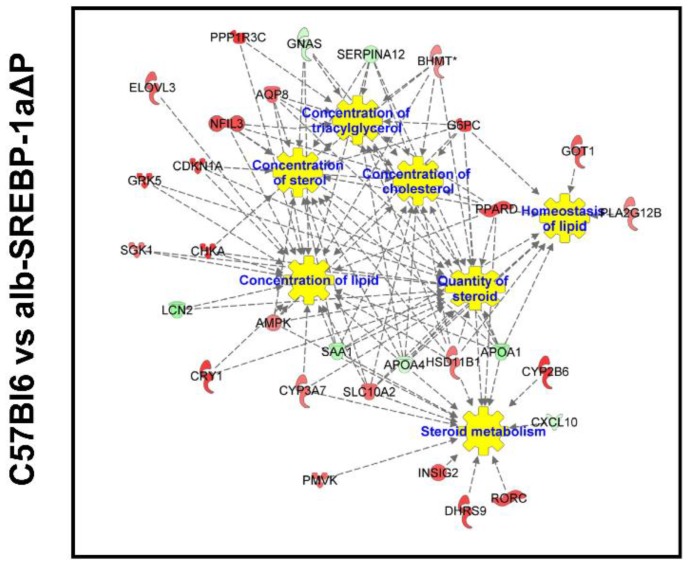
Functional network of SREBP-1-centered subnets. Differential abundant genes of the SREBP-1a subnet, the alb-SREBP-1a∆P subnet, and the phosphorylation subnet were used for IPA Core analyses. Color code indicates: red: increase in condition 1, green: decrease in condition 1 based on measured expression differences. Relation to pathophysiological function is indicated.

We next focused on genes of the SREBP-1 subnetworks (SREBP-1a subnet, SREBP-1a∆P subnet, phosphorylation subnet). Re-analysis of these transcripts in regard to downstream signaling identified an impact on concentration of lipids, sterols, diacylglycerols, and steroids as well as lipid homeostasis as main functions (Figure 6, Table S6). Nevertheless, there were differences according to the SREBP-1a subnet and SREBP-1a∆P subnet. Overall, fewer genes were identified in the phosphorylation-deficient signaling and they differed from the regular SREBP-1a signaling. The direct comparison of differential abundant transcripts of the phosphorylation subnet showed that glucose or energy metabolism and insulin-related signaling as well as cell survival accounted for the highest significances, in contrast to the lipid-centered pathways in the SREBP-1a and SREBP-1a∆P subnet (Table S6).

Next to expression differences observed, genes in the SREBP-1a∆P subnet correlated to clinical measures (BG, insulin sensitivity and β-cell function, SDH activity, cholesterol, TFA, liver weight) in C57Bl6 mice, but the correlation was lost in alb-SREBP-1a∆P mice. In the alb-SREBP-1a∆P mice, we observed correlation to TG, catalase activity, adipose tissue, and liver weight. Nine of these genes, including *Insig2, Elovl3*, and *G6pc*, correlated to specific catalase activity in the functional alb-SREBP-1a. Again, correlation was lost in the phosphorylation-deficient mouse model. One gene (*Lnc2*) showed inverse correlation in alb-SREBP-1a and alb-SREBP-1a∆P mice (Table S7). The phosphorylation subnet consisted of a total of 21 differential regulated transcripts between alb-SREBP-1a and alb-SREBP-1a∆P. Of these, 11 transcripts correlated to catalase activity in the genotypes. Seven of them negatively correlated to catalase activity in functional alb-SREBP-1a, three transcripts in alb-SREBP-1a∆P, or in C57Bl6. The latter correlations were not observed in SREBP-1a mice (Table S7).

### 2.7. Effect of Disruption of SREBP-1a Phosphorylation at MAPK Sites on Peroxisomal Proteome Patterns

Central interaction pathways indicated the molecular impact of intact SREBP-1a regulation by phosphorylation seem to involve ACOX or transcriptional networks especially mediated by PPARa, and the differential gene expression influenced by SREBP-1a phosphorylation pointed to peroxisomal catalase activity. Furthermore, the ability to phosphorylate SREBP-1a at MAPK sites had an impact on peroxisomal function, indicated as catalase activity. The differences in the peroxisomal protein patterns in the comparison of C57Bl6 vs. alb-SREBP-1a mice revealed only 12 differential abundant peroxisomal proteins (two increased in C57Bl6; 10 increased in alb-SREBP-1a) (Supplement Table S8). As the holistic investigation of overall peroxisomal protein alterations due to SREBP-1a phosphorylation was determined in an untargeted 2D-DIGE approach and consecutive mass spectrometry, which allows the direct and quantitative comparability of the experimental conditions, protein patterns were not further validated with another technique. Knowledge-based analyses of the differential abundant proteins are not informative due to the low number of differential proteins. The comparison of C57Bl6 vs. alb-SREBP-1a is summarized in Table S9; examples are given in Table 3. Solely suggested upstream elements INSR, MYC, or PPARA and networks centering around DSP, mediator, FBXO32, FBXW7, or HNF4A showed the highest overlap.

The comparison of C57Bl6 and alb-SREBP-1a∆P identified106 peroxisomal proteins (41 increased in C57Bl6; 67 increased in alb-SREBP-1a∆P). Redundant identification of proteins is immanent to the technique used, and can be due to protein modifications (Table S8). Knowledge-based analyses of the differential abundant proteins of the comparison of C57Bl6 and alb-SREBP-1a∆P are summarized in Table S10; examples are given in Table 3. A significant enrichment of pathways associated with fatty acid β-oxidation, mitochondrial dysfunction, tricarboxylic acid (TCA) Cycle II, amino acid degradation, arginine biosynthesis, sirtuin signaling, oxidative phosphorylation, or urea cycle was obtained. These pathways involve upstream regulators PPARA, PPARG, PPARGC1A, HNF4A, and signaling via INSR, ACOX1, MAP4K4, or LONP1. Suggested functions were centered on fatty acid metabolism or mitochondrial dysfunction, inhibition of RXR function, and PPARα/RXRα activation. The differential abundant proteins were assigned hepatic steatosis, liver proliferation and growth, or nonalcoholic fatty liver disease.

The ability to phosphorylate SREBP-1a further interferes with the peroxisomal protein patterns. The comparison of alb-SREBP-1a and alb-SREBP-1a∆P resulted in 130 differential abundant peroxisomal proteins (36 increased in alb-SREBP-1a; 99 increased in alb-SREBP-1a∆P) (Table S8). Knowledge-based analyses of the differential abundant proteins of the comparison of alb-SREBP-1a vs. alb-SREBP-1a∆P is summarized in Table S11; examples are given in Table 3. Here, in principle the same pathways were identified, but not necessarily the same pathway interacting proteins were differentially abundant. The differential abundant proteins can be assigned to various pathways including proteins ascertain to mitochondrial dysfunction, oxidative phosphorylation, sirtuin signaling, lipid metabolism including fatty acid β-oxidation, branched-chain α-keto acid dehydrogenase complex, 2-oxobutanoate degradation, acetyl-CoA, or fatty acid α-oxidation. Furthermore, proteins could be assigned to TCA Cycle, or amino acid degradation or biosynthesis, and other central metabolic degradation processes (urea cycle, ethanol or glycine betaine degradation, xenobiotic metabolism signaling, as well as PPARα/RXRα activation). Knowledge-based analyses identified the main upstream molecules involved were transcriptional activators PPARA, PPARGC1A, HNF4A, PPARG, KLF15, Esrra, and signaling proteins INSR, MAP4K4, LONP1, LEP, or growth hormone. Central networks involved were centered on INSR, ABHD5, ACOX1, and PPARA.

## 3. Discussion

In the present study, we describe the effect of an inactivation of MAPK-related phosphorylation sites in the central lipid metabolic transcription factor SREBP-1a on hepatic gene expression and peroxisome functionality. We compared mice overexpressing the transcriptional active domain of human SREBP-1a and a previously introduced mouse model that is deficient in MAPK ERK-1/-2, JNK and p38 stress kinase phosphorylation sites (S63A, S117A, T426V) [12]. Although having a similar phenotype as the controls, the observed differences in hepatic gene expression and peroxisomal function were unexpectedly larger in the phosphorylation-deficient SREBP-1a∆P, indicating broad regulatory alterations.

Even though the pathogenesis of NAFLD is still unclear, SREBP-1a appears to be a central player. Previous mechanistically investigations in animal models suggested that the metabolic gene regulatory profile of the liver interferes with environmental factors to determine fatty liver and metabolic syndrome (MetS) pathogenesis [9,19,20,21,22,23].

One major trigger of MetS is obesity, due to an access of systemic lipids presented by overnutrition or dysregulated DNL, fatty acid β-oxidation, lipid transport or clearance. Nevertheless, the risk to develop fatty liver increases with the number of MetS risk components like obesity, dyslipidemia and diabetes focusing on insulin resistance and BG as further important predictor of NAFLD [2,24,25].

The limited alterations in hepatic transcriptome and peroxisomal proteome in alb-SREBP-1a mice might be explained by the overpresence of a SREBP-1a molecule, which can be regulated as the endogenous molecule. Overall, increased mature SREBP-1a levels target regular SREBP-1a-related pathways, but there were also signs of counter regulation, e.g., the high expression of NR1D1, or degradation pathways like SIRT or RNA modification via proteasome for signal ablation. In the phosphorylation-deficient model; however, the same pathways were activated as in alb-SREBP-1a, but, interestingly, different molecules are responsive, indicating that the regulation is somehow spoiled.

NR1D1 (REV-ERB alpha) is phosphorylation-independent and the highest differentially regulated transcript in alb-SREBP-1a and alb-SREBP-1a∆P. It has been shown that NR1D1 interferes with the proteolytic activation of SREBPs in Golgi membranes via Insig2 transcription activation and therefore in sequestering of mature SREBP proteins to the endoplasmic reticulum membranes [26,27]. Of note, Insig2 is downregulated in alb-SREBP-1a, but not in alb-SREBP-1a∆P, a further indication of disturbed regulation processes. NR1D1 also regulates cholesterol-7α-hydroxylase (CYP7A1) expression, the rate-limiting enzyme in converting cholesterol to bile acids [27], making a possible link to the reduced cholesterol levels observed in alb-SREBP-1a∆P.

Another mechanism to attenuate signaling might be highlighted by the overrepresentation of SIRT signaling components. The highly-conserved NAD+ dependent histone and non-histone deacetylase SIRT decreases FA synthesis and increases β-oxidation. Mechanistically this is mediated via generation of *O*-acetyl-ADP-ribose and regulated hormonally or environmentally via cellular NAD+ concentrations. For example, Sirt-1 is involved in NAFLD and steatosis development in humans and mice [28,29,30], where SIRT increase counteracts the fatty liver phenotype.

The vast number of olfactory receptors (OLFR) overrepresented in alb-SREBP-1a∆P is puzzling. OLFR genes are the largest gene family in the eukaryotic genome and they are expressed in olfactory active cells in a “one specific OLFR per cell” fashion to enable adaptive olfaction. The gene regulation of OLFR genes is mainly constitutive with posttranscriptional degradation [31]. There were no hints up to now that this gene family is expressed in the liver to the observed extent. Several OLFR have been identified in non-olfactory tissues, and these interact with metabolism, maybe in “sniffing” available metabolite concentrations for metabolic adaptation and regulation of energy metabolism. These include OLFR that act on hepatic TG or FFA metabolism, adipose tissue FA lipolysis, or beta cell insulin secretion and further evidence is just emerging [32,33,34]. Although the biological relevance is unclear, our study confirms an earlier observation of increased OLFR gene expression in fatty liver of an adipose tissue transplantation model [35]. The emergence of OLFRs is a sign of disturbed gene regulation due to the phosphorylation-deficient SREBP-1a transcription factor, which might function as a dominant negative bait SREBP-1 molecule.

SREBPs act as homo- or heterodimers dimers to activate genes in lipid metabolism, whereas the composition of dimers has different transactivation potential. SREBP-1a as well as SREBP-2 homodimers or a SREBP-1a/2 heterodimers activate target genes more robustly than SREBP-1c homodimer [36]. In addition, the activation domain of both dimerization partners is essential for full transcriptional activity [37]. In this context, our construct might act as homodimer with low transactivity or as a negative bait for hetero-dimerization with the endogenous SREBPs. In regard to hormone regulation of lipid metabolic gene expression, this construct might result in hepatic insulin resistance selectively on lipid metabolism.

Metabolic fitness interferes with the activity of intracellular organelles in healthy and diseased conditions. We and others recently reported that cellular organelles controlling energy homeostasis, i.e., mitochondria and peroxisomes, are involved in the development of fatty liver and the metabolic syndrome [17,18,38,39]. Especially peroxisomes showed up as first aid adaption to altered metabolic needs [18]. In this study, catalase activity was elevated in alb-SREBP-1a∆P, independent of increased liver weight, accompanied by alterations in the peroxisomal proteome. Furthermore, PPARA, HNF4a, or peroxisomal ACOX-1 expression differences were of the highest enrichments indicating the well-characterized integrative expression network of gene expression of peroxisomal target genes [40,41]. In line with a previous report of a protective effect of endogenous catalase activity in fatty liver [42], a further novel finding of our study was the observation that half of the genes that center around SREBP-1 and differ in regulation according to the ability of SREBP-1a to be phosphorylated are correlated with peroxisomal catalase activity. These included genes like CWF19L1, which was previously shown to act in fatty liver and metabolic diseases [43,44,45,46]. Furthermore *Lcn2* (lipocalin-2) correlated to catalase activity dependent on SREBP-1a phosphorylation. In support of our observation, Lcn-2 was shown to influence liver TG and oxidative stress via subcellular organelles [47].

Hepatic insulin resistance is sufficient to induce dyslipidemia as in MetS. In states of insulin resistance plasma glucose is increased, in combination with increased hepatic lipids and plasma TG due to reduced muscle glucose uptake accompanied by increased adipose tissue lipolysis. Mechanistically, increasing BG shifts from glucose production via gluconeogenesis to glycogen storage, with insulin regulating gluconeogenesis and lipogenesis in liver. SREBP-1 integrates several phosphorylation cascades on several regulatory levels. Degradation of active SREBP-1 is controlled by phosphorylation cascades [15]. In regard to insulin signaling, phosphorylation of SREBPs by MAPK couples insulin to the gene regulatory machinery [14]. Insulin signaling involves a phosphorylation cascades with numerous targets. In regard to hepatic glucose metabolism the forkhead transcription factor O1 (FoxO1) is a central regulator and defined endpoint of insulin signaling. Phosphorylation of FoxO1 interferes with its nuclear translocation, so insulin signaling attenuates the transactivation of gluconeogenesis by reduced transcription of PEPCK and G6Pase. In parallel, lipid metabolism is activated by SREBP-1 as a direct target of insulin signaling via MAPK [10]. This activation is completely abolished if the phosphorylation sites were mutated [11,12,13,14]. In this respect, SREBP-1 integrates the hormone signal also to transcriptional activation of DNL and also lipid transport via LDL receptor or VLDL assembling [12,48]. Of note, we identified a SREBP-1 mutation in direct proximity of S117 in a patient with dyslipidemia phenotype and marked hypocholesterinemia [48].

In hepatic insulin resistance, FoxO1 is no longer inhibited and SREBP-1 is active. As a consequence, there is gluconeogenesis and lipogenesis at the same time, resulting in increased glucose and lipid content of the cells. This concept was impressive demonstrated in the liver specific insulin receptor KO (LIRKO) mice, as a genetic model of isolated hepatic insulin resistance [49]. LIRKO mice show reduced liver weight, hyperglycemia, hyperinsulinemia dyslipidemia and hypercholesterinemia accompanied by reduced expression of SREBP family genes and their downstream targets [49]. Our investigations indicate that, in regard to SREBP-1-regulated genes, alb-SREBP-1a∆P mice show a shift from lipid metabolic pathways to insulin resistance, thus uncoupling one metabolic arm of hepatic insulin resistance and resembling the model for the selective versus total insulin resistance suggested by Brown and Goldstein [20].

In Conclusion, like the LIRKO mice, alb-SREBP-1a∆P mice resemble a hepatic model of insulin resistance, but in contrast to LIRKO appears to be selective for lipid metabolism. Compared to the alb-SREBP-1a mice there are phenotypical parallels of alb-SREBP-1a∆P to LIRKO, but, in contrast to LIRKO mice, the alb-SREBP-1a∆P mice showed hypocholesterinemia. This, in combination with the increased peroxisomal activity, might point to the importance of peroxisomal function and its role in the compartmentation of cholesterol and bile acid synthesis.

Taken together, although there is a clear phenotype of mice having abundant SREBP-1a activity, there are no gross alterations compared to wild-type mice at the cellular level regarding hepatic gene expression profiles and proteome of peroxisomes. However, the inability of SREBP-1 phosphorylation to take place by MAPK leads to major changes in cellular signaling cascades, although phenotype per se was almost not affected. This might indicate that post-translational modification of transcription factors does not interfere with the baseline phenotype, but might affect responses to external stimuli, like prevention of fatty liver disease in these mice.

## 4. Materials and Methods 

### 4.1. Animals and Phenotype Characterization

C57Bl6 (C57Bl6), C57Bl6-TgN alb-HA-hSREBP-1a-NT (alb-SREBP-1a) and C57Bl6-TgN alb-HA-hSREBP-1a∆P-NT (alb-SREBP-1a∆P) were used in the study [12]. Mice were bred and maintained under standard conditions (12 h light/dark cycle; 22 ± 1 °C, 50 ± 5% humidity). Male litter mates of each genotype (*n* = 15 each) were kept under standardized conditions with free access to water and regular laboratory chow (13.7 mJ/kg: 53% carbohydrate, 36% protein, 11% fat (Ssniff, Soest, Germany)) from six to 24 weeks of age. Mice were sacrificed by CO_2_ asphyxiation (7:00 a.m.). Blood samples were collected by left ventricular puncture and livers were removed. The Animal Care Committee of the University of Duesseldorf approved animal care and procedure (Approval #50.05-240-35/06, August 2006).

Phenotypical characterization, serum diagnostics of clinical measures, and surrogate parameters of insulin resistance were performed as previously described [12,17,18]. Serum-free fatty acid (FFA) and hepatic total fatty acid (TFA) contents were determined by gas chromatography. Hormone concentrations (insulin, glucagon, leptin, adiponectin, GIP, GLP-1 and ghrelin) in serum were determined with the Multiplex Immunoassay Bioplex System (BioRad, Bio-Plex™ Protein Array System, Munich, Germany) according to the supplier’s instructions.

Peroxisomal proteome analyses, 2D-DIGE™, and protein identification were completed by MALDI-MS.

Subcellular fractionation was used to isolate peroxisomes from 1.5 g fresh liver tissue. The organelle quality of all preparation steps were monitored by marker enzyme activity in isolated organelles, and electron microscopy as described [12,17,18]. Marker enzyme assays and mitochondrial copy number were determined as described [12,47,50]. Highly enriched peroxisomes were processed in 2D-DIGE^™^ experiments.

In brief, per 2D-DIGE gel comparison 50 μg proteins were labeled with 400 pmol cyanine dyes Cy3 and Cy5 (GE Healthcare, Munich, Germany) [50]. Samples were labeled in separate replicates with either Cy3 or Cy5 to avoid dye bias. For each comparison, equal amounts of unlabeled protein (25 µg) were combined as internal standard and labeled with Cy2 dye. Per experiment, proteins of condition 1 (Cy3) and 2 (Cy5; or vice versa) and the Cy2-labeled internal standard were combined and subjected to isoelectric focusing (IEF) on a MultiPhor II electrophoresis unit (Amersham Biosciences, Freiburg Germany) using IPG strips (24 cm; pH 4–7, pH 6–9; linear) according to the manufacturer’s recommendations. IEF-focused proteins were then separated by size in the second dimension on a 1.0 mm thick linear 12% polyacrylamide gels (24 cm × 18 cm) combined with a Tris (0.1 M)/Tricine (0.1 M) buffer system (EttanDalt 12 system (Amersham Biosciences)). Gels were scanned (Typhoon 9400 (Amersham Biosciences) at a resolution of 100 µm, with a photomultiplier tube of 550 V. For spot picking, gels were subsequently stained with colloidal Coomassie. Determination of protein spot abundance and statistics was carried out automatically using Proteomwaver, 4.0, (BioRad, Munich, Germany). The analysis parameters were set to a standardized average spot volume ratio of 1.5-fold, *p* < 0.01, 20% CV, significantly altered protein spots need to be present in *n* = 4 replicates. Differential protein spots were analyzed by MALDI-MS in a time-of-flight Ultraflex-Tof/Tof (BrukerDaltoniks, Bremen, Germany). Proteins were identified using the mouse subset of Swiss-Prot (Sprot_2016 (Available online: http://www.uniprot.org/)) non-redundant database and our peroxisomal database reference maps (Available online: http://www.diabesityprot.org) as described [12,17,18]. Data are available at our organelle database (Available online: http://www.diabesityprot.org).

### 4.2. Gene Expression Analyses

RNA extraction (Qiagen, Hilden, Germany) of snap frozen biopsies were performed as described [18]. Genome wide expression analyses (*n* = 8 per genotype) were performed with 150 ng RNA according to the Ambion WT Expression Kit and the WT Terminal Labelling Kit (Affymetrix, Freiburg, Germany). All protocol steps were monitored using a RNA 6000 nano kit (Agilent, Taufkirchen, Germany). Complementary RNA samples were hybridized to Mouse Gene 1.0 ST arrays, and analyzed with a GeneChip scanner 3000 7G (GDAS 1.4 package, Affymetrix, (Thermofisher Scientific, Darmstadt, Germany)). Data were analyzed with Expression Console^TM^ v1.1 and Transcriptome Analysis Console^TM^ v2.0 (Affymetrix) as described [17]. Full datasets are available under accession number GSE110569 (Available online: www.ncbi.nlm.nih.gov/geo/).

For functional annotation of differentially expressed genes and peroxisomal proteins, Web-based tools from public database sources were used. Information-driven analyses including functional annotation was performed with IPA^®^ (Ingenuity^®^ Pathway Analysis winter release 2017, Qiagen, Hilden, Germany). For gene expression analyses, expression fold change (1.5-fold) and expression differences of the separate comparisons C57Bl6 vs. alb-SREBP-1a, C57Bl6 vs. alb-SREBP-1a∆P and alb-SREBP-1a vs. alb-SREBP-1a∆P (*p*-value < 0.05) were analyzed following the IPA^®^ core analyses modules. The differential peroxisomal protein patterns of the comparisons C57Bl6 vs. alb-SREBP-1a, C57Bl6 vs. alb-SREBP-1a∆P and alb-SREBP-1a vs. alb-SREBP-1a∆P were also analyzed in IPA^®^ using expression fold change and *t*-test derived *p*-values. Pathways were generated form respective networks suggested by IPA^®^.

Statistical analyses were performed in Prism 7.0 (GraphPad Inc, San Diego, USA) and SPSS (version 24 IBM, Boeblingen, Deutschland). Data are given as mean ± standard deviation (SD) and data were directly compared with an unpaired Student’s *t* test. Clinical values are presented as mean ± SD. Multiplex Immunoassay Data were analyzed by Bio-Plex Manager™ (Version 6.1) Software (BioRad, Munich, Germany). Pearson correlation coefficients with a two-tailed *p*-value were determined in SPSS.

## Figures and Tables

**Table 1:** Differentially abundant transcripts in the comparisons C57Bl6 vs. alb-SREBP-1a, C57Bl6 vs. alb-SREBP-1a∆P and alb-SREBP-1a vs. alb-SREBP-1a∆P. The numbers of differential regulated transcripts in the comparisons C57Bl6 vs. alb-SREBP-1a, C57Bl6 vs. alb-SREBP-1a∆P, and alb-SREBP-1a vs. alb-SREBP-1a∆P and the top 15 up and down regulated molecules with highest significance are shown (only annotated transcripts, negative value: more abundant in condition 1, positive value: more abundant in condition 2. Complete analyses are given in Table S2). Abbreviations are: ANOVA, analysis of variance, FDR, fals discovery rate.

**C57Bl6 vs. alb-SREBP-1a**					
**All (*n*)**	**Unique Transcripts (*n*)**	**More Abundant in C57Bl6 (*n*)**	**More Abundant in alb-SREBP-1a (*n*)**	**Unknown Function (*n*)**	**Not Annotated (*n*)**
**794**	**586**	**169**	**437**	**250**	**63**
**Fold Change (Linear)**	**ANOVA *p*-Value**	**FDR *p*-Value**	**Gene Symbol**	**Main Description**	
−9.35	1.56 × 10^−12^	4.52 × 10^−8^	*Nr1d1*	nuclear receptor subfamily 1, group D, member 1	
−2.34	8.09 × 10^−11^	1.00 × 10^−6^	*Mtmr9*	myotubularin-related protein 9	
−1.92	3.05 × 10^−9^	9.00 × 10^−6^	*Cspp1*	centrosome and spindle pole associated protein 1	
−2.05	3.55 × 10^−9^	9.00 × 10^−6^	*Eif4ebp2*	eukaryotic translation initiation factor 4E binding protein 2	
−2.8	1.24 × 10^−8^	1.40 × 10^−5^	*Arrdc3*	arrestin domain containing 3	
−1.96	1.32 × 10^−8^	1.40 × 10^−5^	*Fcer1g*	Fc receptor, IgE, high affinity I, gamma polypeptide	
−126.7	1.46 × 10^−8^	1.40 × 10^−5^	*mt-Ts2*	mitochondrially encoded tRNA serine 2	
−1.84	1.48 × 10^−8^	1.40 × 10^−5^	*Hcfc1r1*	host cell factor C1 regulator 1 (*XPO1*-dependent)	
−1.92	1.85 × 10^−8^	1.70 × 10^−5^	*Krtcap2*	keratinocyte associated protein 2	
−114.04	2.19 × 10^−8^	1.70 × 10^−5^	*mt-Ty*	mitochondrially encoded tRNA tyrosine	
−1.88	2.66 × 10^−8^	1.90 × 10^−5^	*Laptm5*	lysosomal-associated protein transmembrane 5	
−1.93	4.28 × 10^−8^	2.20 × 10^−5^	*Cd52*	CD52 antigen	
−1.9	4.49 × 10^−8^	2.20 × 10^−5^	*Ear10*	eosinophil-associated, ribonuclease A family, member 10	
−2.14	4.92 × 10^−8^	2.30 × 10^−5^	*C1qc*	complement component 1, q subcomponent, C chain	
−1.92	6.31 × 10^−8^	2.70 × 10^−5^	*Mn1*	meningioma 1	
1.68	6.87 × 10^−9^	1.00 × 10^−5^	*Ahcy*	S-adenosylhomocysteine hydrolase	
2.02	9.74 × 10^−9^	1.30 × 10^−5^	*Ppard*	peroxisome proliferator activator receptor delta	
1.59	2.82 × 10^−8^	1.90 × 10^−5^	*Sfxn1*	sideroflexin 1	
1.59	4.53 × 10^−8^	2.20 × 10^−5^	*Ivd*	isovaleryl coenzyme A dehydrogenase	
1.55	6.83 × 10^−8^	2.80 × 10^−5^	*Nfil3*	nuclear factor, interleukin 3, regulated	
1.55	1.03 × 10^−7^	3.60 × 10^−5^	*Pttg1ip*	pituitary tumor-transforming 1 interacting protein	
1.55	1.03 × 10^−7^	3.60 × 10^−5^	*Hspd1*	heat shock protein 1 (chaperonin); predicted gene 12141	
1.97	1.69 × 10^−7^	5.00 × 10^−5^	*Acnat1*	acyl-coenzyme A amino acid *N*-acyltransferase 1	
2.75	2.20 × 10^−7^	5.80 × 10^−5^	*Snora69*	small nucleolar RNA, H/ACA box 69	
1.75	2.93 × 10^−7^	7.00 × 10^−5^	*Hacl1*	2-hydroxyacyl-CoA lyase 1	
1.53	3.60 × 10^−7^	8.00 × 10^−5^	*Bcap31*	B cell receptor associated protein 31	
1.92	4.28 × 10^−7^	8.90 × 10^−5^	*Tmem254b*	transmembrane protein 254b	
1.8	4.64 × 10^−7^	9.40 × 10^−5^	*Tat*	tyrosine aminotransferase	
1.81	5.69 × 10^−7^	1.11 × 10^−4^	*Fga*	fibrinogen alpha chain	
1.58	5.96 × 10^−7^	1.13 × 10^−4^	*Spop*	speckle-type POZ protein	
**C57Bl6 vs. alb-SREBP-1a ΔP**					
**All (*n*)**	**Unique Transcripts (*n*)**	**More Abundant in C57Bl6 (*n*)**	**more Abundant in alb-SREBP-1a (*n*)**	**Unknown Function (*n*)**	**not Annotated (*n*)**
**4581**	**3723**	**1732**	**1991**	**950**	**370**
**Fold Change (Linear)**	**ANOVA *p*-Value**	**FDR *p*-Value**	**Gene Symbol**	**Main Description**	
−108.6	1.92 × 10^−14^	2.78 × 10^−10^	*mt-Ts2*	mitochondrially encoded tRNA serine 2	
−11.38	1.09 × 10^−14^	2.78 × 10^−10^	*Nr1d1*	nuclear receptor subfamily 1, group D, member 1	
−187.66	6.92 × 10^−14^	6.67 × 10^−10^	*mt-Tt*	mitochondrially encoded tRNA threonine	
−96.52	1.31 × 10^−13^	9.45 × 10^−10^	*mt-Ty*	mitochondrially encoded tRNA tyrosine	
−15.04	2.01 × 10^−12^	1.16 × 10^−8^	*mt-Tp*	mitochondrially encoded tRNA proline	
−2.32	4.57 × 10^−12^	2.21 × 10^−8^	*Rnasek*	ribonuclease, RNase K	
−3.02	2.57 × 10^−11^	5.32 × 10^−8^	*Snord57*	small nucleolar RNA, C/D box 57; NOP56 ribonucleoprotein	
−20.09	4.35 × 10^−11^	7.86 × 10^−8^	*mt-Tk*	mitochondrially encoded tRNA lysine	
−13.23	7.61 × 10^−11^	1.10 × 10^−7^	*mt-Tn*	mitochondrially encoded tRNA asparagine	
−1.81	8.65 × 10^−11^	1.19 × 10^−7^	*Vmn1r170*	vomeronasal 1 receptor 170	
−1.75	3.34 × 10^−10^	1.91 × 10^−7^	*Gsdmc2*	gasdermin C2; gasdermin C3; gasdermin C4	
−3.31	2.94 × 10^−10^	1.91 × 10^−7^	*Mir692-1*	microRNA 692-1	
−4.02	6.36 × 10^−10^	2.25 × 10^−7^	*Dbp*	D site albumin promoter binding protein	
−3.18	6.82 × 10^−10^	2.32 × 10^−7^	*mt-Te*	mitochondrially encoded tRNA glutamic acid	
−8.36	1.41 × 10^−9^	3.90 × 10^−7^	*mt-Tc*	mitochondrially encoded tRNA cysteine	
1.58	7.82 × 10^−12^	2.52 × 10^−8^	*Timm23*	translocase of inner mitochondrial membrane 23	
1.78	5.46 × 10^−10^	2.00 × 10^−7^	*Adck3*	aarF domain containing kinase 3	
1.86	4.19 × 10^−10^	1.91 × 10^−7^	*Mrpl36*	mitochondrial ribosomal protein L36	
1.9	7.46 × 10^−11^	1.10 × 10^−7^	*Hsd11b1*	hydroxysteroid 11-beta dehydrogenase 1	
1.93	2.84 × 10^−10^	1.91 × 10^−7^	*Sgpl1*	sphingosine phosphate lyase 1	
1.98	1.93 × 10^−10^	1.91 × 10^−7^	*Spop*	speckle-type POZ protein	
2.11	1.26 × 10^−10^	1.66 × 10^−7^	*Bcap31*	B cell receptor associated protein 31	
2.4	4.19 × 10^−10^	1.91 × 10^−7^	*Tubb2a*	tubulin, beta 2A class IIA	
2.43	2.23 × 10^−11^	4.96 × 10^−8^	*Ppard*	peroxisome proliferator activator receptor delta	
2.57	2.09 × 10^−10^	1.91 × 10^−7^	*Snord104*	small nucleolar RNA, C/D box 104	
3.56	1.96 × 10^−10^	1.91 × 10^−7^	*Npas2*	neuronal PAS domain protein 2	
1.51	1.40 × 10^−9^	3.90 × 10^−7^	*G3bp1*	GTPase activating protein (SH3 domain) binding protein 1	
1.53	2.81 × 10^−9^	6.41 × 10^−7^	*Rbmxl1*	RNA binding motif protein	
1.55	3.46 × 10^−9^	7.10 × 10^−7^	*Nubp2*	nucleotide binding protein 2	
1.57	3.01 × 10^−9^	6.74 × 10^−7^	Sumo2	small ubiquitin-related modifier 2-like	
**alb-SREBP-1a vs. alb-SREBP-1aΔP**					
**All (*n*)**	**Unique Transcripts (*n*)**	**More Abundant in C57Bl6 (*n*)**	**More Abundant in alb-SREBP-1a (*n*)**	**Unknown Function (*n*)**	**Not Annotated (*n*)**
**3385**	**2544**	**1143**	**1401**	**276**	**930**
**Fold Change (Linear))**	**ANOVA *p*-Value**	**FDR *p*-Value**	**Gene Symbol**	**Main Description**	
−1.98	6.42 × 10^−9^	7.10 × 10^−5^	*Xlr5a*	X-linked lymphocyte-regulated 5A, pseudogene	
−1.66	7.17 × 10^−8^	8.20 × 10^−5^	*Olfr1410*	olfactory receptor 1410	
−1.91	8.85 × 10^−8^	8.20 × 10^−5^	*Olfr796*	olfactory receptor 796	
−2.04	2.14 × 10^−7^	9.50 × 10^−5^	*Olfr154*	olfactory receptor 154	
−1.84	2.37 × 10^−7^	9.50 × 10^−5^	*Olfr169*	olfactory receptor 169	
−2.06	2.52 × 10^−7^	9.50 × 10^−5^	*Olfr644*	olfactory receptor 644	
−1.54	2.97 × 10^−7^	9.80 × 10^−5^	*Slc12a8*	solute carrier family 12, member 8	
−2	3.24 × 10^−7^	1.02 × 10^−4^	*Vmn1r193*	vomeronasal 1 receptor 193	
−2.52	3.40 × 10^−7^	1.04 × 10^−4^	*Vmn1r37*	vomeronasal 1 receptor 37pseudogene 21	
−1.7	5.92 × 10^−7^	1.24 × 10^−4^	*Olfr1269*	olfactory receptor 1269	
−2.15	6.13 × 10^−7^	1.24 × 10^−4^	*Krtap10-10*	keratin associated protein 10-10	
−1.84	8.08 × 10^−7^	1.30 × 10^−4^	*Clps*	colipase, pancreatic	
−2.49	8.93 × 10^−7^	1.37 × 10^−4^	*Mir301b*	microRNA 301b	
−2.34	9.64 × 10^−7^	1.42 × 10^−4^	*Olfr488*	olfactory receptor 488	
−1.57	1.00 × 10^−6^	1.56 × 10^−4^	*Adamts17*	a disintegrin-like and metallopeptidase thrombospondin type 1 motif, 17	
1.6	1.59 × 10^−8^	7.10 × 10^−5^	*Pde8a*	phosphodiesterase 8A	
2.3	3.24 × 10^−8^	7.10 × 10^−5^	*Ppp2r5c*	protein phosphatase 2, regulatory subunit B, gamma	
1.9	3.46 × 10^−8^	7.10 × 10^−5^	*Qpct*	glutaminyl-peptide cyclotransferase	
1.86	3.60 × 10^−8^	7.10 × 10^−5^	*Slirp*	SRA stem-loop interacting RNA binding protein	
1.77	3.66 × 10^−8^	7.10 × 10^−5^	*Asf1a*	anti-silencing function 1A histone chaperone	
1.65	4.60 × 10^−8^	7.60 × 10^−5^	*Mrpl36*	mitochondrial ribosomal protein L36	
1.66	6.08 × 10^−8^	8.20 × 10^−5^	*Psma5*	proteasome (prosome, macropain) subunit, alpha type 5	
1.61	7.01 × 10^−8^	8.20 × 10^−5^	*Syne2*	spectrin repeat containing, nuclear envelope 2	
1.62	7.94 × 10^−8^	8.20 × 10^−5^	*Usp40*	ubiquitin specific peptidase 40	
1.75	8.06 × 10^−8^	8.20 × 10^−5^	*Gtf2a2*	general transcription factor II A, 2	
1.64	9.07 × 10^−8^	8.20 × 10^−5^	*Nt5c2*	5-nucleotidase, cytosolic II	
1.54	1.01 × 10^−7^	8.60 × 10^−5^	*Pccb*	propionyl Coenzyme A carboxylase, beta polypeptide	
1.62	1.20 × 10^−7^	8.60 × 10^−5^	*Pdzd11*	PDZ domain containing 11	
1.62	1.22 × 10^−7^	8.60 × 10^−5^	*Lig3*	ligase III, DNA, ATP-dependent	
1.75	1.23 × 10^−7^	8.60 × 10^−5^	*Acadl*	acyl-Coenzyme A dehydrogenase, long-chain	

**Table 2:** Differential expression Core analyses of regulated transcripts in the comparisons *C57Bl6* vs. *alb-SREBP-1a*, *C57Bl6* vs. *alb-SREBP-1a∆P* and *alb-SREBP-1a* vs. *alb-SREBP-1a∆P*. Examples as listed in text. *p*-value: *p*-value for enrichment of pathway molecules in the data set. Complete analyses are given in Tables S3–S5.

**C57Bl6 vs. alb-SREBP-1a**							
**Canonical Pathways**	***p*-Value**	**Upstream Activator**	***p*-Value**	**Disease or Tox Function**	***p*-Value**	**Interaction Network**	***p*-Value**
FXR/RXR	6.76 × 10^−6^	RORA	2.67 × 10^−18^	hepatic steatosis associated pathways.	1.60 × 10^−7^	ACOX1	1.76 × 10^−12^
PPAR	1.58 × 10^−4^	RORC	1.98 × 10^−13^	liver cholestasis	1.30 × 10^−3^	MAPK7	2.93 × 10^−12^
sirtuin signaling	2.63 × 10^−4^	PPARA	1.22 × 10^−11^	liver hyperplasia/hyperproliferation	2.76 × 10^−3^	ONECUT1	3.16 × 10^−11^
		PPARD	1.16 × 10^−8^	liver proliferation	1.80 × 10^−3^	PPARA	3.33 × 10^−9^
		GPD1	5.31 × 10^−11^	renal damage	6.15 × 10^−4^		
		SLC25A13	7.24 × 10^−11^	nonalcoholic fatty liver disease	8.13 × 10^−3^		
		HNF4A	4.59 × 10^−6^				
**C57Bl6 vs. alb-SREBP-1a ΔP**							
**Canonical Pathways**	***p*-Value**	**Upstream Activator**	***p*-Value**	**Disease or Tox Function**	***p*-Value**	**Interaction Network**	***p*-Value**
methionine degradation	3.24 × 10^−6^	HNF4A	9.23 × 10^−29^	olfactory response	2.32 × 10^−144^	ACOX1	2.51 × 10^−9^
isoleucinedegradation	3.89 × 10^−4^	RORC	1.56 × 10^−9^	olfaction	1.68 × 10^−139^	GRB14	6.14 × 10^−9^
valine degradation	2.40 × 10^−3^	RORA	7.56 × 10^−9^	signal transduction	4.91 × 10^−60^	PPARGC1A	2.10 × 10^−6^
cysteine biosynthesis	9.33 × 10^−5^	ONECUT1	2.85 × 10^−8^	Cell communication	1.30 × 10^−55^		
autophagy	1.12 × 10^−3^	MYC	4.47 × 10^−7^	RNA post-transcriptional modification	2.08 × 10^−6^		
protein ubiquitination	1.12 × 10^−3^	NR1D1	1.76 × 10^−6^	synthesis of ribonucleoside monophosphate	7.74 × 10^−6^		
sirtuin signaling	8.51 × 10^−3^	ACOX1	4.78 × 10^−10^	amino acids metabolism	6.02 × 10^−10^		
ceramide biosynthesis	4.27 × 10^−3^	PPARA	6.16 × 10^−6^	oxidation of lipid	3.08 × 10^−6^		
fatty acid β-oxidation	2.29 × 10^−2^	RXRA	8.74 × 10^−4^	oxidation of fatty acids	8.63 × 10^−6^		
				fatty acid metabolism	9.55 × 10^−6^		
				bleeding of liver	1.55 × 10^−3^		
				compensated cirrhosis	7.29 × 10^−3^		
				liver degeneration	7.31 × 10^−3^		
				cholestasis	8.51 × 10^−3^		
**alb-SREBP-1a vs. alb-SREBP-1aΔP**							
**Canonical Pathways**	***p*-Value**	**Upstream Activator**	***p*-Value**	**Disease or Tox Function**	***p*-Value**	**Interaction Network**	***p*-Value**
RNA polymerase II complex assembly	9.33 × 10^−5^	HNF4A	4.34 × 10^−13^	olfactory response	7.51 × 10^−123^	NCAM1	2.17 × 10^−5^
protein ubiquitination	9.12 × 10^−4^	RORC	6.11 × 10^−5^	olfaction	2.92 × 10^−120^	ACOX1	4.32 × 10^−5^
sirtuin signaling	1.02 × 10^−2^	RORA	6.26 × 10^−5^	signal transduction	7.55 × 10^−61^	SIRT4	1.56 × 10^−4^
sumoylation	3.72 × 10^−2^	PPARA	2.13 × 10^−4^	cell communication	1.49 × 10^−57^	NR2F1	2.55 × 10^−4^
		ACOX1	1.71 × 10^−5^	RNA post-transcriptional processing	2.69 × 10^−7^	FOXO4	4.82 × 10^−4^
				RNA transport	2.66 × 10^−6^	FABP1	5.98 × 10^−4^
				differentiation processes	1.78 × 10^−5^	ABCB1	9.88 × 10^−4^
				clearance of bilirubin	6.17 × 10^−3^	PPARA	1.17 × 10^−3^
				proliferation of hepatic stellate cells	4.42 × 10^−2^		
				hepatomegaly	6.43 × 10^−2^		

**Table 3:** Differential peroxisomal proteome and core analyses of the comparisons C57Bl6 vs. alb-SREBP-1a, C57Bl6 vs. alb-SREBP-1a∆P and alb-SREBP-1a vs. alb-SREBP-1a∆P. Examples as listed in text. *p*-value: *p*-value for enrichment of pathway molecules in the data set. Complete analyses are given in Tables S9–S11.

**C57Bl6 vs. alb-SREBP-1a**							
**Canonical Pathways**	***p*-Value**	**Upstream Activators**	***p*-Value**	**Disease, Function**	***p*-Value**	**Interaction Networks**	***p*-Value**
		INSR	9.95 × 10^−7^			DSP	1.23 × 10^−8^
		MYC	1.00 × 10^−5^			mediator	4.45 × 10^−8^
		PPARA	7.24 × 10^−5^			FBXO32	3.95 × 10^−7^
						FBXW	4.0 × 10^−7^
						HNF4A	5.46 × 10^−7^
**C57Bl6 vs. alb-SREBP-1a ΔP**							
**Canonical Pathways**	***p*-Value**	**Upstream Activators**	***p*-Value**	**Disease, Function**	***p*-Value**	**Interaction Networks**	***p*-Value**
fatty acid β-oxidation	3.98 × 10^−16^	PPARA	2.67 × 10^−34^	microvesicular hepatic steatosis	1.21 × 10^−17^	INSR	9.63 × 10^−30^
mitochondrial dysfunction	1.585 × 10^−13^	PPARG	1.64 × 10^−12^	hepatic steatosis	1.2 × 10^−16^	ACOX1	1.64 × 10^−14^
TCA Cycle II	1.585 × 10^−11^	PPARGC1A	2.05 × 10^−11^	liver hyperplasia/hyperproliferation	5.33 × 10^−4^	MAP4K4	7.86 × 10^−13^
valine degradation	2.45 × 10^−10^	HNF4A	3.67 × 10^−13^	nonalcoholic fatty liver disease	0.000106	LONP1	4.36 × 10^−11^
Methionine degradation	9.55 × 10^−7^			fatty acid metabolism	7.94 × 10^−24^		
arginine biosynthesis	8.91 × 10^−9^			mitochondrial dysfunction	1.99 × 10^−13^		
sirtuin signaling	1.99 × 10^−9^			inhibition of RXR function	4.89 × 10^−7^		
oxidative phosphorylation	3.72 × 10^−9^			PPARα/RXRα activation	3.31 × 10^−5^		
urea cycle	2.4 × 10^−6^						
**alb-SREBP-1a vs. alb-SREBP-1aΔP**							
**Canonical Pathways**	***p*-Value**	**Upstream Activators**	***p*-Value**	**Disease, Function**	***p*-Value**	**Interaction Networks**	***p*-Value**
mitochondrial dysfunction	2.51 × 10^−26^	PPARA	8.36 × 10^−34^	microvascular hepatic steatosis	9.98 × 10^−15^	INSR	3.25 × 10^−38^
oxidative phosphorylation	1.26 × 10^−19^	PPARGC1A	1.38 × 10^−18^	liver growth regulation	1.24 × 10^−6^	ABHD5	7.00 × 10^−35^
sirtuin signaling	1.58 × 10^−19^	HNF4A	9.90 × 10^−14^	nonalcoholic fatty liver disease	1.16 × 10^−5^	ACOX1	2.57 × 10^−31^
fatty acid β-oxidation	2.51 × 10^−15^	PPARG	5.48 × 10^−13^	TCA Cycle	6.31 × 10^−13^	PPARA	3.60 × 10^−31^
branched-chain α-keto acid dehydrogenase complex	8.71 × 10^−7^	KLF15	4.72 × 10^−12^	valine degradation	7.94 × 10^−12^		
2-oxobutanoate degradation	2.14 × 10^−6^	Esrra	1.26 × 10^−11^	methionine degradation	1.82 × 10^−9^		
acetyl-CoA oxidation	7.41 × 10^−6^	INSR	2.07 × 10^−28^	citrulline degradation	2.14 × 10^−8^		
fatty acid α-oxidation	2.29 × 10^−4^	MAP4K4	5.20 × 10^−19^	Isoleucine biosynthesis	1.23 × 10^−6^		
		LONP1	1.17 × 10^−16^	arginine biosynthesis	1.91 × 10^−8^		
		LEP	1.37 × 10^−11^	urea cycle	1.91 × 10^−8^		
		growth hormone	5.60 × 10^−11^	ethanol degradation	2.34 × 10^−7^		
				glycine betaine degradation	2.63 × 10^−7^		
				xenobiotic metabolism signaling	1.38 × 10^−6^		
				PPARα/RXRα activation	1.00 × 10^−4^		

## Supplementary Materials

Supplementary materials can be found at http://www.mdpi.com/1422-0067/19/4/980/s1.

## Abbreviations

ABCB1	ATP-binding cassette, sub-family B (MDR/TAP), member 1
ABDH5	1-acylglycerol-3-phosphate O-acyltransferase
ACOX1	acyl-Coenzyme A oxidase 1
DUS	desmoplakin
ERK	mitogen-activated protein kinase 1
Essra	estrogen receptor releated alpha
FABP1	fatty acid binding protein 1
FBXO32	F-box protein 32
FBXW	F-box and WD repeat containing
FOXO4	forkhead box O4
FXR	nuclear receptor subfamily 1, group H, member 4
GPD1	glycerol-3-phosphate dehydrogenase 1
GPD1	hexose-6-phosphate dehydrogenase
GRB14	growth factor receptor bound protein 14
GSK3	glycogen synthase kinase 3 beta
HNF4A	hepatic nuclear factor 4, alpha
INSR	insulin receptor
JNK	mitogen-activated protein kinase 8
KLF15	Kruppel-like factor 15
Lcn2	lipocalin 2
LDL	low density lipoprotein
LEP	leptin
LONP1	lon peptidase 1
LXR	Liver X receptor
MAPK	mitogen-activated protein kinase
MAPKAP4	mitogen-activated protein kinase activating protein 4
MYC	myelocytomatosis oncogene
NCAM1	neural cell adhesion molecule 1
NR1D1	nuclear receptor subfamily 1, group D, member 1
NR2F1	nuclear receptor subfamily 2, group F, member 1
NR2F1	nuclear receptor subfamily 2, group F, member 2
ONECUT1	one cut domain, family member 1
PPARA	peroxisome proliferator activated receptor alpha
PPARD	peroxisome proliferator activator receptor delta
PPARGC1A	peroxisome proliferative activated receptor, gamma, coactivator 1 alpha
RORA	retinoic acid receptor (RAR)-related orphan receptor alpha
RORC	retinoic acid receptor (RAR)-related orphan receptor gamma
RXR	retinoid x receptor
SIRT4	sirtuin 4
SLC25A13	solute carrier family 25 member 13
TCA	tricarboxylic acid
VLDL	very low density lipoprotein
